# Improved targeting of an anti‐TAG‐72 antibody drug conjugate for the treatment of ovarian cancer

**DOI:** 10.1002/cam4.3078

**Published:** 2020-05-05

**Authors:** Megan Minnix, Lin Li, Paul Yazaki, Junie Chea, Erasmus Poku, David Colcher, John E. Shively

**Affiliations:** ^1^ Department of Molecular Imaging and Therapy Beckman Research Institute City of Hope Duarte CA USA; ^2^ Irell and Manella Graduate School of Biological Sciences Beckman Research Institute City of Hope Duarte CA USA; ^3^ Radiopharmacy City of Hope Medical Center Duarte CA USA

**Keywords:** antibody drug conjugate, ovarian cancer, TAG72

## Abstract

**Introduction:**

Ovarian cancer has only a 17% 5‐year survival rate in patients diagnosed with late stage disease. Tumor‐associated glycoprotein‐72 (TAG72), expressed in 88% of all stages of ovarian cancer, is an excellent candidate for antibody‐targeted therapy, as it is not expressed in normal human adult tissues, except in the secretory endometrium.

**Methods:**

Using the clinically relevant anti‐TAG72 murine monoclonal antibody CC49, we evaluated antibody drug conjugates (ADCs) incorporating the highly potent, synthetic antimitotic agent monomethylauristatin E (MMAE). MMAE was conjugated to CC49 via reduced disulfides in the hinge region, using three different types of linker chemistry, vinylsulfone (VS‐MMAE), bromoacetamido (Br‐MMAE), and maleimido (mal‐MMAE).

**Results:**

The drug antibody ratios (DARs) of the three ADCs were 2.3 for VS‐MMAE, 10 for Br‐MMAE, and 9.5 for mal‐MMAE. All three ADCs exhibited excellent tumor to blood ratios on PET imaging, but the absolute uptake of CC49‐mal‐MMAE (3.3%ID/g) was low compared to CC49‐Br‐MMAE (6.43%ID/g), at 142 hours. Blood clearance at 43 hours was 38% for intact CC49, about 24% for both CC49‐VS‐MMAE and CC49‐Br‐MMAE, and 7% for CC49‐mal‐MMAE. CC49‐VS‐MMAE was not further studied due to its low DAR, while CC49‐mal‐MMAE was ineffective in the OVCAR3 xenograft likely due to its rapid blood clearance. In contrast, CC49‐Br‐MMAE treated mice exhibited an average of a 15.6 day tumor growth delay and a 40% increase in survival vs controls with four doses of 7.5 or 15 mg/kg of CC49‐Br‐MMAE.

**Conclusion:**

We conclude that CC49‐Br‐MMAE with a high DAR and stable linker performs well in a difficult to treat solid tumor model.

## Graphical abstract

Antibody drug conjugates with three different linker chemistries were evaluated for targeting TAG72 positive xenografts in an ovarian cancer model. An ADC with ten bromoacetamido linked monomethylauristatin drugs per antibody performed best in terms of tumor targeting and therapy.

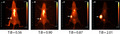

## 1 INTRODUCTION

Ovarian cancer is the fifth most common cause of death in women due to cancer, with minimal improvement in first‐line therapies.^1^ More than 70% of patients will relapse after first‐line treatment of surgery and chemotherapy^2^ with less than a 20% survival rate in patients diagnosed with the later stage diseases. In an effort to improve clinical outcomes for ovarian cancer, antibody based, targeted therapies offer the ability to deliver agents directly to the tumor and minimize off‐target toxicity. TAG72, an under glycosylated mucin epitope, stands out among potential ovarian tumor antigens for antibody‐based therapy. Overexpression of TAG72 occurs in 88% of all stages of ovarian cancer with a good correlation between expression and patient prognosis, while normal TAG72 expression is limited to endometrial tissues during the secretory phase.^3^, ^4^, ^5^, ^6^, ^7^ Importantly, radiolabeled B72.3, a first generation anti‐TAG72 specific monoclonal antibody was approved for imaging ovarian tumors as the product Oncoscint.^8^ CC49 a second generation, TAG72 specific monoclonal antibody, recognizes an epitope comprising both carbohydrates and protein,^4^ but has no antitumor activity of its own, unlike some anti‐mucin antibodies that target purely carbohydrate epitopes.^9^ To overcome this limitation, beta‐emitting radionuclide‐labeled CC49 agents have been used in several radioimmunotherapy (RIT) trials, but results were disappointing due to lack of significant clinical responses and/or dose limiting bone marrow toxicity.^10^, ^11^, ^12^ In this study, we investigated alternative cytotoxic payloads attached to CC49 in order to improve upon the in vivo efficacy and to maintain high tumor targeting.

Antibody drug conjugates (ADCs), tumor specific antibodies covalently linked to cytotoxic payloads, are an alternate approach to radioimmunotherapy. Their success depends on four factors: choice of antigen and antibody for tumor specificity, and choice of linker and payload for drug delivery. Among the various drug payloads tested, monomethyl Auristatin E (MMAE) has been a popular choice due to its potent antitumor effects.^13^, ^14^ MMAE is an antimitotic agent that inhibits cell division by blocking the polymerization of tubulin. Extensive work has demonstrated that site‐specific conjugation along with appropriate linker choice conjugations allows for a controlled amount of drug to antibody attachment and release without compromising tumor targeting.^15^, ^16^ There are currently four FDA‐approved ADCs, all of which use releasable chemistry for their drug to antibody linkers.^17^, ^18^, ^19^, ^20^ Although only one ADC, Lifastuzumab vedotin, has made it to clinical trials in the treatment of ovarian cancer, it was subsequently discontinued due to insufficient progression free survival.^21^ Thus, further development of ADCs for ovarian cancer is an unmet need.

In an OVCAR3 murine model of ovarian cancer we have tested the efficacy of MMAE attached to reduced disulfides in the hinge region of the murine monoclonal antibody CC49 via three linkers that exhibit different levels of drug attachment and release. We found that the linker with the most stable chemical attachment of the drug to the antibody exhibited the best in vitro and in vivo cytotoxicity and antitumor effects.

## 2 MATERIALS AND METHODS

### 2.1 Reagents

MMAE was from MedChemExpress. Divinyl sulfone, Tris (2‐carboxyethyl)phosphine (TCEP) and maleimide were from Sigma‐Aldrich. Bromoacetic anhydride was from Fisher Scientific. DOTA‐NHS‐ester was from Macrocyclics, Inc. The murine CC49 hybridoma clone was obtained through ATCC (HB‐9459™) and antibody produced in the Theranostic Center at City of Hope. Purification was performed as previously described.^22^ The murine CC49 antibody showed a purity of >98% by SDS gel electrophoresis. SDS gel electrophoresis was also used to assess purity of ADCs. The daratumumab (Dara) antibody was from Janssen Biotech Inc.

### 2.2 Chemistry

#### 2.2.1 Synthesis of vinyl sulfone‐MMAE

Divinyl sulfone (VS, 2.09 mmol, 217.5 µL) and MMAE (150 mg, 0.209 mmol) were refluxed for 2 days in 12 mL dry MeOH plus catalytic amounts of Indium (lll) Chloride (InCl_3_) (4.62 mg, 0.0209 mmol). MeOH was evaporated, the crude product dissolved in 5 mL of acetonitrile and purified on a Gemini 5uC18 110A RP column (Phenomenex) using a gradient of 5% to 95% of 90% Acetonitrile/0.1% Trifluoroacetic acid (Buffer B) on an AKTA chromatography system (GE Life Sciences), with the product peak eluting at 69.4% Buffer B. The yield of purified product was 92.7 mg (53.1%). The molecular weight of the product was 836.14 as determined by ESI/MS on a Thermo Finnigan LTQ mass spectrometer.

#### 2.2.2 Synthesis of bromoacetamido‐MMAE

Bromoacetic anhydride (217 mg, 0.836 mmol) was reacted with MMAE (300 mg, 0.418 mmol) in 1.5 mL of dry DMF under argon overnight at 25°C and purified on Gemini RP column above using a gradient of 5%‐95%, with product peak eluting at 90.2% Buffer B. The yield of purified product was 291 mg (82.9%). The molecular weight of the product was 838.93 as determined by ESI/MS on a Thermo Finnigan LTQ mass spectrometer.

#### 2.2.3 Synthesis of maleimido‐MMAE

Maleimide (5.31 mg, 0.0547 mmol) was reacted with bromoacteamido‐MMAE (22.92 mg, 0.0274 mmol) in 0.5 mL DMF in the presence of solid NaHCO_3_ (23.0 mg, 0.274 mmol). The product was purified using the Gemini RP column above using a gradient of 0%‐100%, with product peak eluting at 91.8% Buffer B, to give 13.82 mg (35.1% yield) of product. The molecular weight of the product was 855.09 as determined by ESI/MS on a Thermo Finnigan LTQ mass spectrometer.

#### 2.2.4 Antibody conjugation to vinyl sulfone‐MMAE

CC49 (8.0 mg, 53 nmole) in 1.6 mL of PBS, 1.0 mmol/L EDTA, pH 7.5, was reduced with a 30 molar excess of TCEP for 2 hours at 37°C. TCEP was removed with a 7K MWCO Zeba Spin Desalting column (Thermo Scientific). The vinyl sulfone‐MMAE (VS‐MMAE) drug‐linker (1.34 mg, 1.60 µmole) was rapidly added to the reduced antibody, and reacted overnight at 25°C. The percent yield of ADC was 78%, as determined by absorbance at 280 nm.

#### 2.2.5 Antibody conjugation to bromoacetamido‐MMAE

CC49 (41.8 mg, 279 nmol) in 4.0 mL of PBS, 1.0 mmol/L EDTA, pH 7.5, was reduced with 30 molar excess of TCEP for 2 hours at 37°C. TCEP was removed with a 7K MWCO Zeba Spin Desalting column. The bromoacetamido‐MMAE (Br‐MMAE) drug‐linker (7.02 mg, 8.37 μmol) was rapidly added to the reduced antibody, and reacted overnight at 25°C. Dara, an anti‐CD38 antibody, was used as an untargeted control. Dara (20 mg, 133 nmol) in 1.12 mL of PBS, 1.0 mmol/L EDTA, pH 7.5, was reduced with 30 molar excess of TCEP for 2 hours at 37°C. TCEP was removed with a 7K MWCO Zeba Spin Desalting column. The Br‐MMAE drug‐linker (2.24 mg, 2.67 μmol) was rapidly added to the reduced antibody, and reacted overnight at 25°C. The percent yield was 71%, as determined by absorbance at 280 nm.

#### 2.2.6 Antibody conjugation to maleimido‐MMAE

CC49 (14.9 mg, 99.7 nmol) in 1.32 mL of PBS, 1.0 mmol/L EDTA, pH 7.5, was reduced with 30 molar excess of TCEP for 2 hours at 37°C. TCEP was removed with a 7K MWCO Zeba Spin Desalting column. The maleimido‐MMAE (mal‐MMAE) drug‐linker (2.56 mg, 3 μmol) was rapidly added to the reduced antibody, and reacted overnight at 25°C. The percent yield was 81%, as determined by absorbance at 280 nm.

#### 2.2.7 Determination of the drug antibody ratio (DAR)

DAR was determined using QTOF mass spectrometry (Agilent Technology 6510 QTOF LC/MS). Conjugated antibody (0.6 µg, 40 pmol) in 20 µL PBS was reduced with a 30 molar excess of TCEP for 2 hours at 37°C. The DAR of CC49‐VS‐MMAE was calculated as 2.27. The DAR of CC49‐Br‐MMAE was calculated as 10.0. The DAR of Dara‐Br‐MMAE was calculated as 10.0. The DAR of CC49‐mal‐MMAE was calculated as 9.54.

#### 2.2.8 Radiolabeling studies

CC49 was directly radiolabeled with Iodine‐124 (^124^I) (3D Imaging, Maumelle, AR), onto antibody tyrosine's, at a labeling ratio of 10 μCi/μg. Radiolabeled antibodies were purified by SEC 10/30 GL column (GE Biosciences).^23^ Radiolabel yields were between 70% and 100% as determined by ITLC.

### 2.3 Cell lines and culture

Both OVCAR3 and HT‐29 were obtained from ATCC. Cell lines were cultured at 37°C with 5% CO_2_ in ATCC‐recommended media with 10%‐20% heat inactivated fetal bovine serum (FBS; Corning) supplemented with 2 mmol/L L‐glutamine (L‐glut; MP Biomedicals LLC). OVCAR3 was grown in RPMI 1640 (Corning), 20% FBS, and L‐glut. HT‐29 was grown in McCoy's 5A (ATCC), 10% FBS, and L‐glut. Cells were maintained at optimal confluency. All cell lines, periodically tested, were negative for mycoplasma contamination, using the MycoAlert Mycoplasma Detection Kit from Lonza. The OVCAR3 cell line was sent in and cell line validated by ATCC with the Short Tanden Repeat (STR) profiling test (Manassas, VA).

### 2.4 In vitro Studies

#### 2.4.1 Confocal immunofluorescence microscopy

Cells were grown at 37°C for 24 hours on sterile glass cover slips, washed with 0.1 mol/L HEPES buffer and fixed with ice‐cold methanol for 2 minutes at 25°C. To prevent nonspecific binding, cells were then blocked with 10% goat serum in PBS with 0.05% TWEEN for 30 minutes at 25°C. Blocking solution was removed and the slides were incubated with muCC49, diluted in PBS (1:200) for 1 hour at 25°C. Cover slips were then washed 4x with PBS/TWEEN. Goat anti‐mouse Alexa 555 antibody (ThermoFisher) was added (1:200) and the slide incubated 30 minutes at 25°C in the dark. Cover slips were washed 5x with PBS/TWEEN and mounted onto glass slides with an antifade vector shield, containing DAPI. The slides were imaged on the Zeiss LSM880 confocal microscope.

#### 2.4.2 Cytotoxicity analysis

Cytotoxicity was measured by the Vybrant MTT Cell Proliferation assay kit (ThermoFisher). Cells were plated on day 0, in a 96‐well clear bottom plate, and grown in normal media. Twenty‐four hours later, the antibody alone or the ADC was added at 6.25, 12.5, or 25 μg/mL and incubated for 24, 48, and 72 hours. At the collection time points, growth media containing the antibody or ADC was aspirated, 10 μL of 12 mmol/L MTT stock solution added per well and incubated at 37°C for 3 hours. The MTT solution was removed, 100 μL DMSO added to each well and incubated for 10 minutes at 37°C. Absorbance was measured at 540 nm on a CLARIOstar (MBG LabTech).

### 2.5 In vivo studies

#### 2.5.1 Animal studies

All animal handling was done in accordance with IACUC protocol 14043 approved by the City of Hope Institutional Animal Care and Use Committee. Immune deficient NOD.Cg‐Prkdc^scid^Il2rg^tm1Wjl^/SzJ (NSG; Jackson Laboratory) were used. The OVCAR3 cell line was injected subcutaneously (SQ), 1.5 x 10^6^ cells per mouse in PBS. The tumor size was followed by caliper measurements (TS = 0.5(LxW^2^)). Toxicity was measured by monitoring weight loss of the mouse in which >20% weight loss was considered an endpoint. The OVCAR3 SQ tumors developed cystic sacs around the solid tumor. The fluid was collected with an 18 gauge needle and analyzed separately.

#### 2.5.2 Immunohistochemistry

Immunohistochemistry (IHC) was carried out by the pathology core at City of Hope using the Ventana Discovery Ultra autostainer (Ventana Medical Systems, Roche Diagnostics) and the ChromoMap DAB detection system according to manufacturer's recommendations. Briefly, the tissue samples were collected and fixed in 10% Formalin for 2 days and then stored in 70% EtOH. The sampled were blocked in paraffin, sectioned at a thickness of 5 μm and put on positively charged glass slides. Deparaffinization, rehydration, endogenous peroxidase activity inhibition, and antigen retrieval were all performed by the automated stainer. The muCC49 primary antibody was titrated by twofold serial dilutions. After optimization, cell conditioning solution (pH 8.5) at 64 minutes and antibody at 1:100 dilution conditions were used for all of the samples. An ovary cancer tissue array sample was included as a positive control for each run. Following the primary antibody incubation, the secondary antibodies, DISCOVERY anti‐mouse HQ and DISCOVERY anti‐HQ HRP, were added. The staining was visualized with the DISCOVERY ChromoMap DAB Detection system and counterstained with hematoxylin (Ventana). The fluid collected from the tumor was pelleted, fixed in 10% Formalin, washed with PBS, and then stored in 70% EtOH. For fluid staining, the EtOH was aspirated and the pellet blocked in paraffin. The rest of the analysis was carried out as described above.

#### 2.5.3 PET imaging and biodistribution studies

For all ^124^I PET and blood clearance studies, mice were given Potassium Iodide Oral Solution (SSKI) 24 hours prior to radiolabeled antibody injection to block thyroid uptake of metabolized iodine. Tumor‐bearing mice were given an intravenous injection of 1 mg of Immune Globulin (IVIg; Grifols Therapeutics Inc) 2 hours prior to tail vein injection with the radiolabeled antibody conjugate (100 μCi/ mouse). Mice were anesthetized with isoflurane and imaged at 4, 24, and 46 hours with the small‐animal PET scanner (microPET model R4: Siemens/CTIMI). Data were sorted and images reconstructed as previously described.^24^, ^25^ Terminal biodistribution studies were carried out at the end of the PET studies. The mice were euthanized, necropsy performed, and the organs were collected, weighed, and counted for radioactivity on the 1480 WIZARD 3” Automatic Gamma Counter (PerkinElmer). For blood clearance studies, blood was collected by intravenous tail bleeds and counted for radioactivity on the 1480 WIZARD 3” Automatic Gamma Counter. All radioactivity data were corrected for background and radioactive decay from the time of injection, reported as percent counts per minute (CPM) or percent injected dose per gram (%ID/g).

#### 2.5.4 Therapy studies

Therapy was performed on established tumors (50‐100 mm^3^). All mice were randomly assigned into treatment groups in a way that the mean tumor size for each group was comparable. The mice were treated four times with vehicle control (saline), muCC49 antibody alone, or the ADCs CC49‐mal‐MMAE (malADC) or CC49‐Br‐MMAE (BrADC). The animals were dosed twice weekly for 2 weeks, via intraperitoneal (IP) injection. All doses were made up to 200 μL volume with sodium chloride 0.9% (APP Pharmaceuticals LLC). Survival was defined as the time at which the tumor size quadrupled from the original tumor size.

### 2.6 Statistical analysis

One‐way ANOVA (Tukey's multiple comparison test) was used to analyze the tumor growth curves, using Prism 7.02 (GraphPad Software). The test compared each group to the untreated saline control group. The log‐rank Mantel‐Cox test was used to analyze the survival curves, using Prism 7.02 (GraphPad Software). The Mantel‐Cox test gives equal weight to all time points in the survival curve. Each treated group was compared to the saline control group, unless otherwise stated. Differences were considered significant if *P* ≤ .05. Survival was defined as the time ( x ) in which the tumor had quadrupled its original tumor size before treatment (*y*). The correlating *x*‐value was calculated based on the linear slope between the two points ((*x*_1_, *y*_1_) and (*x*_2_, *y*_2_)) around the tumor size at quadruple the original tumor size, using the formula.

$$x=x_{1}+\frac{\left(x_{2}-x_{1}\right)-\left(y_{2}-y_{1}\right)}{\left(y_{2}-y_{1}\right)}$$

## 3 RESULTS

### 3.1 ADC Synthesis

**FIGURE 1:**
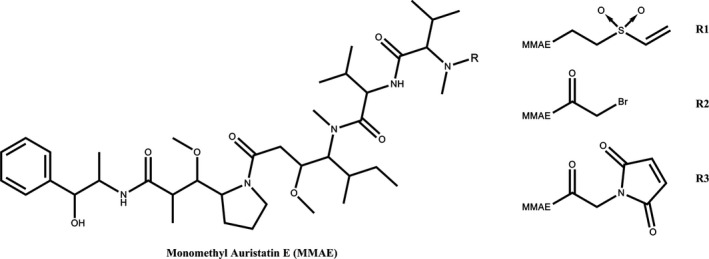
Structure of MMAE and Linkers. R1, Vinyl sulfone linker; R2, Bromoacetamido linker; R3, maleimide linker

Three drug‐linkers were conjugated to the muCC49 antibody on reduced disulfides in the hinge region (Figure 1). In each case, the linkers were conjugated to the amino‐terminus of MMAE at a 30 fold molar excess to mildly reduced CC49 antibody. The purity of the resulting ADCs, as determined by SDS gel electrophoresis, was >95% (Figure S1). The drug‐to‐antibody ratios (DARs) for the three ADCs were 2.3 for VS‐MMAE, 10 for Br‐MMAE, and 9.5 for mal‐MMAE. The low DAR for VS‐MMAE may be due to the less reactive nature of the vinyl sulfone, especially in the context of MMAE (Figures S1‐S4).

### 3.2 In vitro cytotoxicity of the ADCs

**FIGURE 2:**
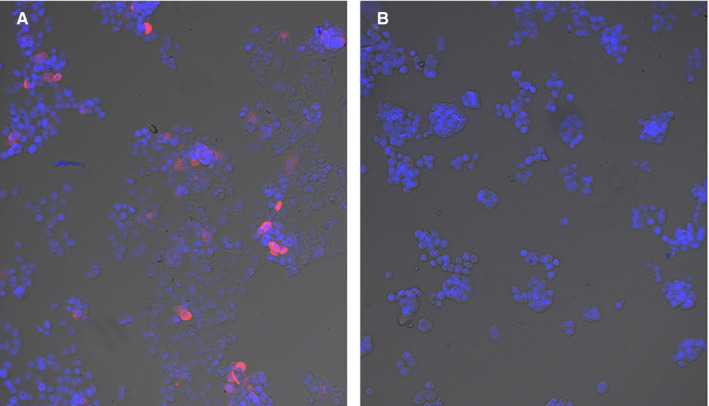
TAG72 expression in OVCAR3 cells using confocal microscopy. OVCAR3 (A) or HT‐29 (B) cells were stained with the CC49 antibody followed by fluorescent labeled rabbit anti‐mouse antibody (red) and DAPI (blue). HT‐29 cells were used as a negative control. Magnification 10x

TAG72 expression was confirmed in the ovarian cell line OVCAR3 by confocal microscopy (Figure 2) with human colorectal carcinoma cell line HT‐29 used as a negative control, since it does not express TAG72.^26^ As expected, TAG72 expression was detected in OVCAR3 but not in HT‐29 cells. Since TAG72 expression in OVCAR3 was found in both the cytoplasm and at the cell surface, these cells serve as an excellent test for the effect of linker of ADC cytotoxicity.

**FIGURE 3:**
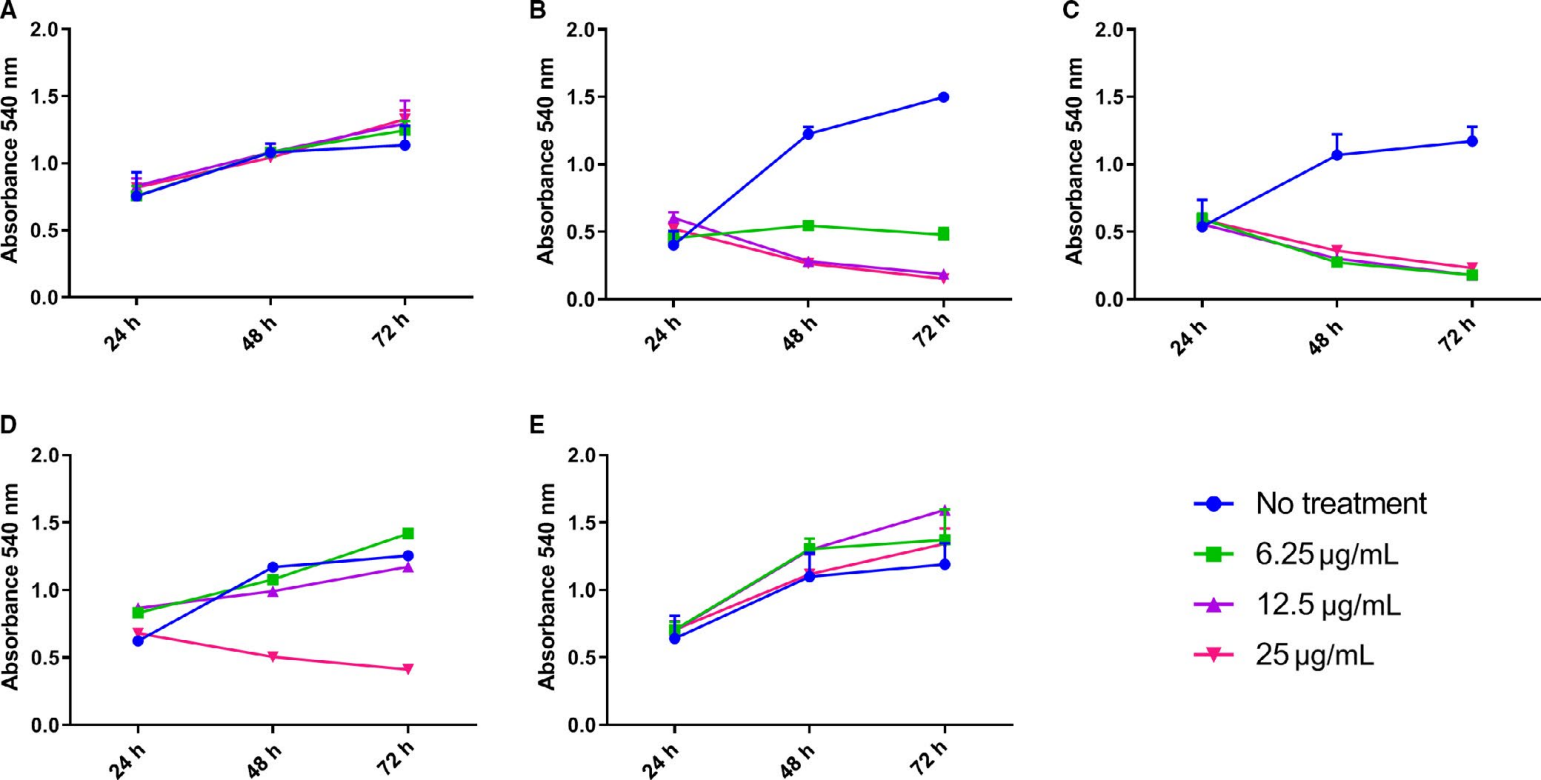
Cytotoxicity of ADCs to OVCAR3 cells. CC49 antibody alone (A), CC49‐VS‐MMAE (B), CC49‐Br‐MMAE (C), CC49‐mal‐MMAE (D), or Dara‐Br‐MMAE (E), were incubated with the OVCAR3 cell line with the indicated doses of ADCs and cytotoxicity measured at the indicated time points (n = 3). Error bars not shown are less than the height of the symbol. Anti‐CD38 (Dara) ADC was used as a negative control

The MTT assay that measures cell metabolic activity, was used to determine ADC mediated cell killing.^27^ In this assay all three CC49 ADCs exhibited a significant dose dependent cell killing at the highest dose, compared to antibody alone at 48 hours (Figure 3). Among the three CC49 ADCs, maleimido‐MMAE had the lowest efficacy, with cytotoxicity observed only at the highest concentration tested. No cytotoxicity was seen with the Dara‐Br‐MMAE, which was used as a nonspecific ADC control, since OVCAR3 cells do not express CD38. Together, these findings indicate a dose‐dependent effect of the CC49 ADCs and target specific cytotoxicity.

### 3.3 In vivo expression of TAG72

**FIGURE 4:**
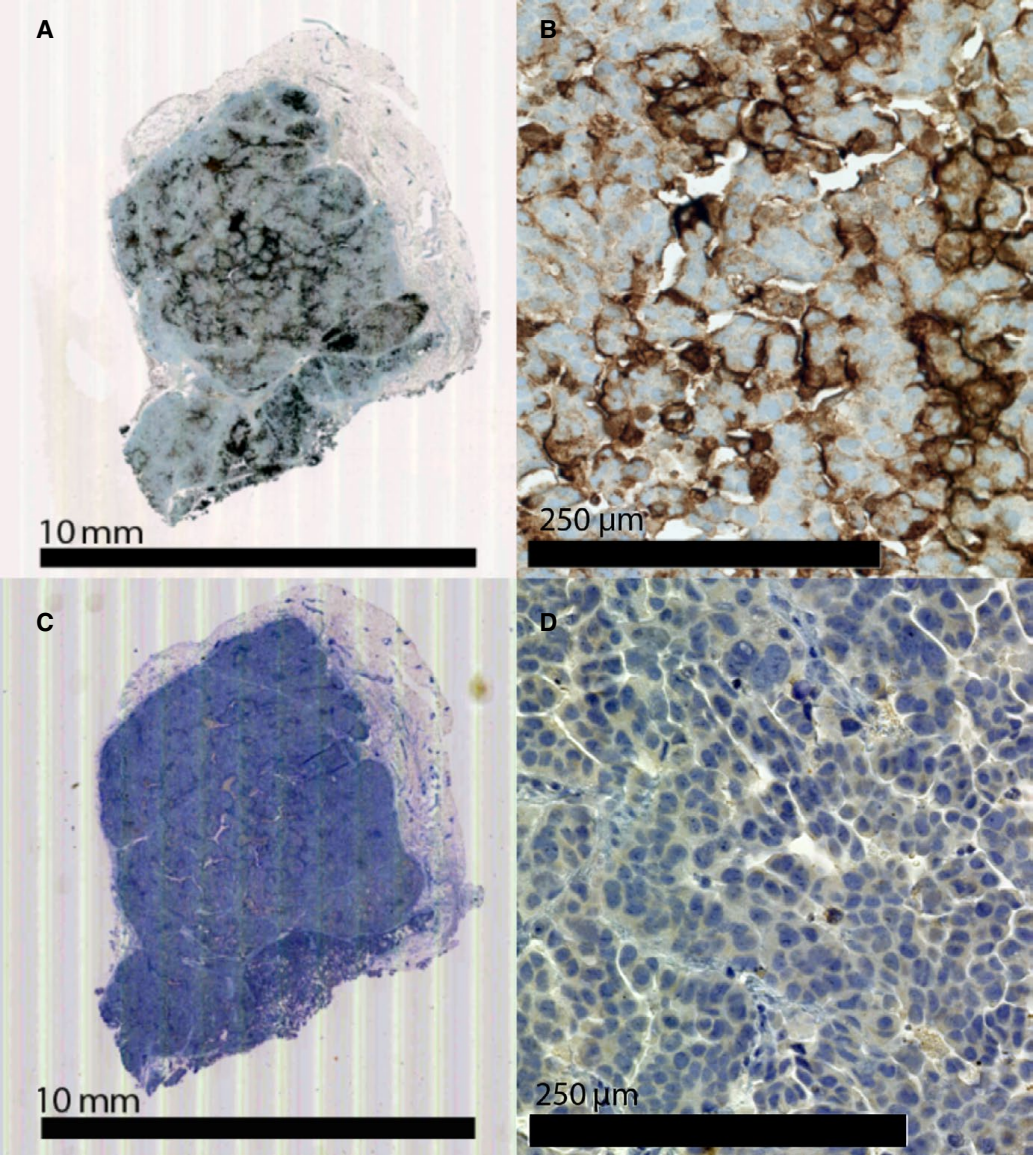
TAG72 Expression in OVCAR3 xenografts. OVCAR3 xenografts in NSG mice were formalin fixed and immunostained with anti‐TAG72 antibody CC49 (A) and (B), or control anti‐CD38 antibody (C) and (D)

In vivo studies were conducted in NSG mice, as these mice allow for rapid tumor engraftment of most human cell lines.^29^ Subcutaneous (SQ) tumors were grown to allow accurate quantitation of tumor size during treatment. Immunostaining of established tumors revealed high levels of TAG72 expression (Figure 4). Immunostaining for CD38 was used as a negative control. In addition these tumors developed fluid filled tumors, partially mimicking the behavior of human ovarian tumors that produce ascites fluid in the IP cavity.^30^ The tumor cystic fluid, formed in the OVCAR3 tumors, also exhibited high levels of TAG72 expression (Figure S5). The uterus and ovaries of the NSG mice were positive when stained with the CC49 antibody (Figure S6). This result is not unexpected since TAG72 is known to be expressed in human endometrial tissue.^31^

### 3.4 PET imaging and pharmacokinetics of the ADCs

**FIGURE 5:**
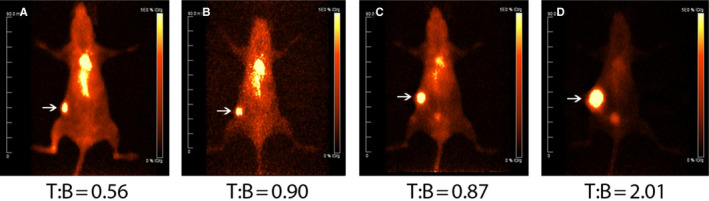
Tumor targeting of OVCAR3 xenografts in NSG female mice with ^124^I‐CC49. PET images of ^124^I‐CC49 (A), ^124^I‐CC49‐VS‐MMAE (B), ^124^I‐CC49‐Br‐MMAE (C), and ^124^I‐CC49‐mal‐MMAE (D) in female NSG mice bearing OVCAR3 tumor at 43‐46 h post‐radiolabeled antibody injection (2 mice/group). Tumors indicated with arrows. Tumor to blood ratios (T:B) are displayed underneath

In order to perform PET imaging, each of the ADCs were radiolabeled with ^124^I. PET imaging was used to compare tumor targeting and determine the tumor to blood ratios of the parent muCC49 antibody vs the CC49 ADCs (Figure 5). All three ADCs were able to target the TAG72 positive OVCAR3 tumors. The CC49 antibody had a tumor to blood ratio of 0.56, 43 hours post radioiodinated antibody injection, while CC49‐VS‐MMAE, CC49‐Br‐MMAE, and CC49‐mal‐MMAE had tumor to blood ratios of 0.90 at 46 hours, 0.87 at 43 hours, and 2.01 at 46 hours, respectively. The terminal biodistributions of the iodinated muCC49 antibody alone and its ADCs are comparable to the blood clearance observed (Figure S7). The antibody alone stays in circulation longer than the ADCs, with a tumor to blood ratio of 1.1 at 142 hours, while the CC49‐Br‐MMAE has a ratio of 2.0 and the muCC49‐mal‐MMAE ratio was 5.9. Although CC49‐mal‐MMAE has a higher tumor to blood ratio, it also exhibited the lowest absolute tumor uptake, at 142 hours, of 3.3%ID/g, likely due to its short half‐life. In comparison the absolute tumor uptake of CC49‐Br‐MMAE at 142 hours was 6.43%ID/g.

**FIGURE 6:**
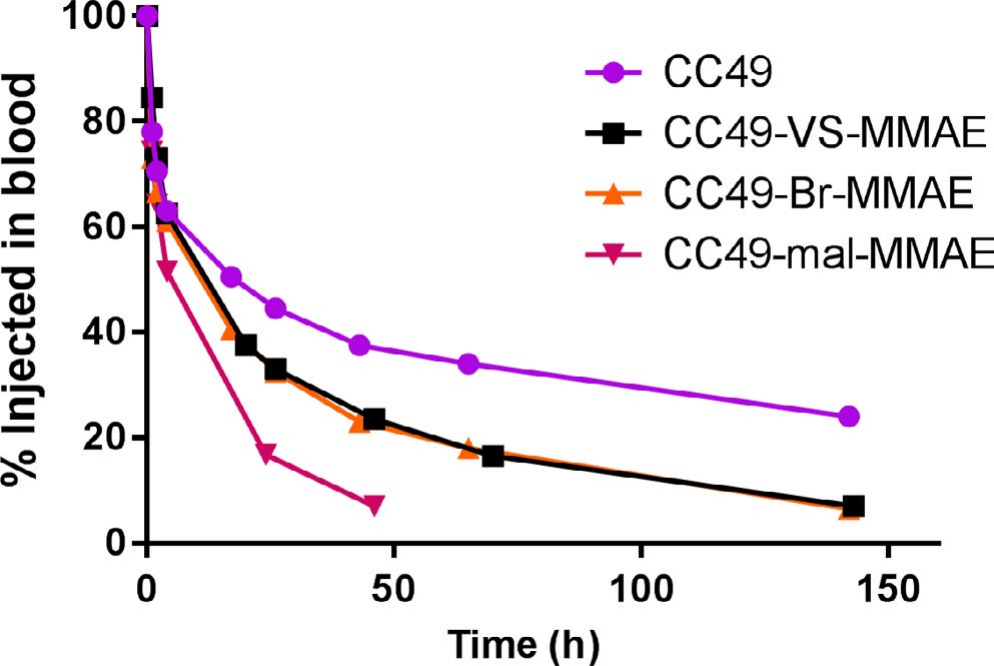
Blood Clearance of CC49 antibody and ADCs in NSG mice. Kinetics of 124I labeled CC49 antibody and ADCs in blood of OVCAR3 xenografted female NSG mice. Percent injected antibody determined by counts in blood at various indicated time points compared to initial counts at time 0 (n = 2/group)

CC49 exhibited slow blood clearance, normal for an intact IgG, with a half‐life of 17.7 hours (Figure 6). CC49‐VS‐MMAE and CC49‐Br‐MMAE had nearly identical pharmacokinetics with a half‐life of 12.0 and 11.0 hours, respectively, despite having differing DARs. CC49‐mal‐MMAE exhibited rapid clearance, with a half‐life of 4.86 hours, despite having the similar DAR of 9.5 as the DAR 10 CC49‐Br‐MMAE. Thus, it appears that the amount of drug loaded onto the antibody is less important than the linker chemistry used. The more stable linkers result in slower clearance, when compared to an ADC using the maleimide linker that can undergo hydrolysis.

### 3.5 In vivo efficacy of the ADCs

**FIGURE 7:**
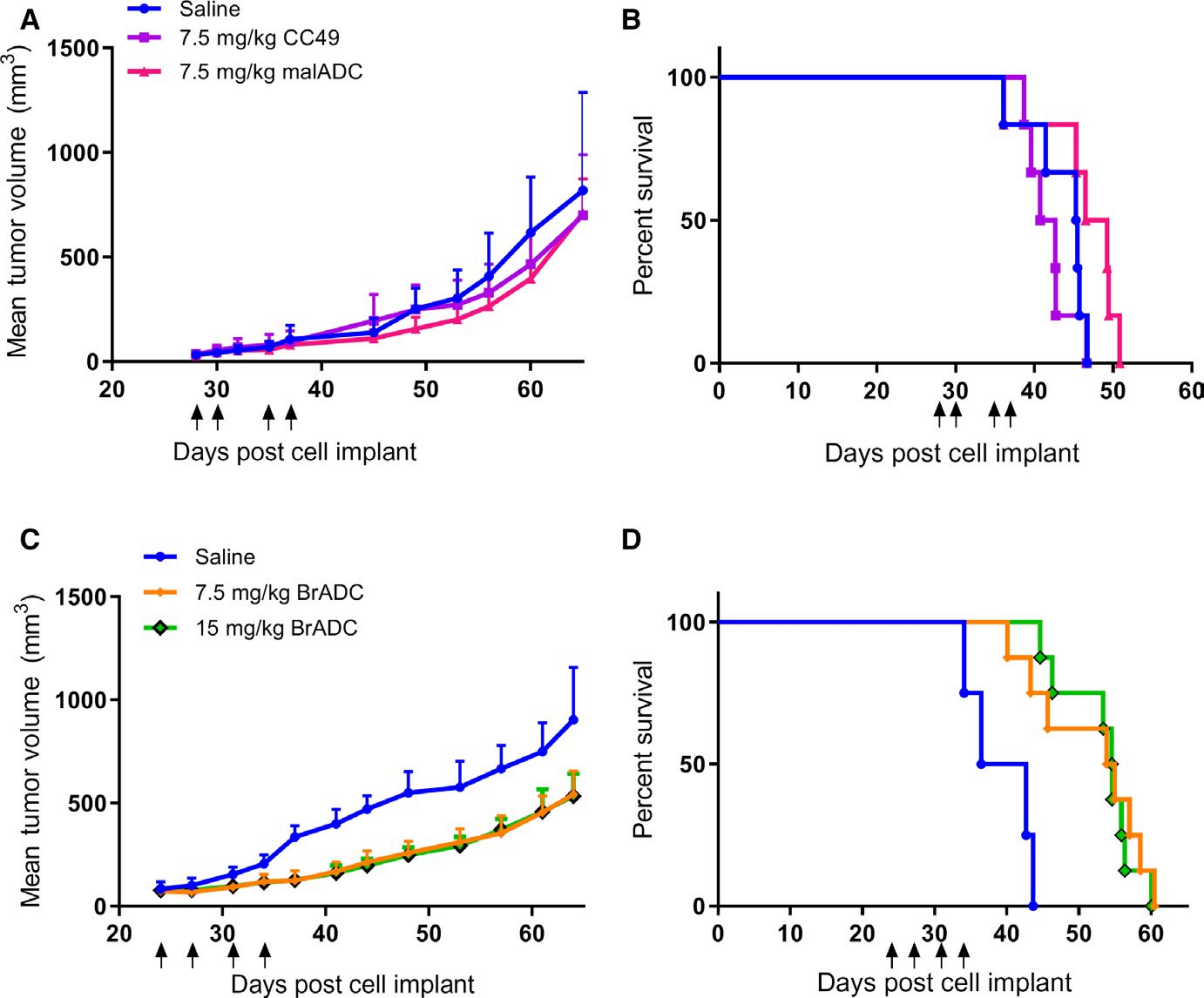
Efficacy of CC49 ADCs in an OVCAR3 xenograft model. Female NSG mice bearing SQ OVCAR3 xeongrafts were treated with 4 IP doses of indicated ADCs. CC49‐mal‐MMAE treated tumors, growth (A) and survival (B) curves at days 28, 30, 35, 37 post OVCAR3 injection. Saline controls (n = 8), CC49 controls (n = 10), and CC49‐mal‐MMAE (n = 10). The tumor growth curves of the antibody alone (*P* = .955) and CC49‐mal‐MMAE (*P* = .764) were not statistically significant compared to the saline control. The survival curves of the antibody alone (*P* = .337) and the CC49‐mal‐MMAE (*P* = .146) were not statistically significant compared to the saline control. CC49‐Br‐MMAE tumor growth (C) and survival (D) curves treated at days 24, 27, 31, and 34 post OVCAR3 injection. Saline (n = 4), 7.5 mg/kg CC49‐Br‐MMAE (n = 8), 15 mg/kg CC49‐Br‐MMAE (n = 8). The tumor growth curves of the 7.5 mg/kg CC49‐Br‐MMAE (*P* = .046*) and 15 mg/kg CC49‐Br‐MMAE (*P* = .044*) were statistically significant compared to the saline control. The survival curves of the 7.5 mg/kg CC49‐Br‐MMAE (*P* = .0064**) and 15 mg/kg CC49‐Br‐MMAE (*P* = .0002***) were statistically significant compared to the saline control. There was no statistical significance between the 7.5 mg/kg CC49‐Br‐MMAE and 15 mg/kg CC49‐Br‐MMAE groups (*P* = .690). Black arrows represent time of injected doses. Statistics: one‐way ANOVA for growth curves and log‐rank test for survival curves

Since CC49‐mal‐MMAE and CC49‐Br‐MMAE had equivalent DARs but different pharmacokinetics, these two ADCs were chosen for analysis of their in vivo antitumor activity in a subcutaneous ovarian cancer OVCAR3 tumor model. This study was also chosen to directly compare the difference between stable and unstable linker chemistries with the same drug and antibody in an animal model. Although CC49‐VS‐MMAE had a similar pharmacokinetics to the bromoacetamido‐MMAE ADC, its low DAR (2.3) suggested it would be a poor choice for therapy. Four doses of the two ADCs were investigated to optimize dosing strategy, with each group receiving doses twice weekly for 2 weeks. The effect of treatment on tumor growth and survival of ADC‐treated mice compared to the vehicle control or the unconjugated antibody‐treated mice is shown in Figure 7. The CC49 antibody alone had no significant effect on either tumor growth or survival. The 7.5 mg/kg doses of the maleimido‐MMAE ADC had a minimal effect on tumor growth rate, with no significant tumor response compared to the control groups (Figure 7A) and no significant effect on survival (Figure 7B). In contrast, bromoacetamido‐MMAE ADC had a significant effect on tumor growth rate and survival at both doses of 7.5 and 15 mg/kg, with a *P* < .05 compared to control (Figure 7C‐D). Both dosage amounts of the CC49‐Br‐MMAE, 7.5 and 15 mg/kg, had similar efficacy, suggesting that the 7.5 mg/kg dose had already reached a maximum effect. Lower doses (1.5 and 3.5 mg/kg) of bromoacetamido‐MMAE ADC were also investigated, but had no significant improvement in efficacy compared to both saline and unconjugated antibody controls (Figure S8). No significant in vivo toxicity for any ADCs was observed, as measured by percent weight loss (Figure S9). Taken together, these data demonstrate that CC49‐Br‐MMAE has a dose‐dependent antitumor effect on ovarian tumors, while the maleimide linked ADC did not show any tumor response. Thus, the more stable linkage using bromoacetamido chemistry improved the efficacy of the DAR 10 ADCs, compared to an unstable linker.

## 4 DISCUSSION

In this study, we were especially interested in treating ovarian cancer, a major malignancy with an unmet need for new and more effective therapies. CC49, like a number of tumor targeting antibodies, has no cytotoxic activity on its own, thus requiring conjugation to a cytotoxic agent for its use in the clinic. The murine CC49 antibody was chosen due to its preclinical history, for its clinical use in imaging and Radioimmunotherapy.^32^, ^33^ It is recognized that a humanized version of CC49 should be investigated in the future, since human anti‐mouse antibodies developed in patients treated with the murine CC49 for Radioimmunotherapy. The generation of ADCs has focused on three parameters, (a) the site of linker‐drug conjugation to the antibody, (b) optimization of the number of linker‐drugs conjugated per antibody, and (c) the stability of the linker under physiological conditions. In terms of site of antibody conjugation, many studies demonstrate conjugation to reduced disulfides in the hinge region preserves antigen binding, offers orthogonal chemistry, and allows for attachment of multiple drugs due to the number of disulfides present in the hinge. Importantly, hinge disulfides can be reduced to free cysteine's under mild conditions without disturbing the many interchain disulfides in antibodies.^36^, ^37^ Thus, we also utilized the reduced hinge as our site of linker‐drug conjugation. Since murine CC49 is an IgG1 with 5 disulfide bonds in its hinge region, a maximum of 10 drugs can be attached to a hinge region reduced antibody. The choice of MMAE as the cytotoxic agent is based on the idea that while this highly toxic agent cannot be used in a monotherapy setting, it has been used successfully as an ADC payload with a number of antibodies,^34^, ^35^ suggesting that it can be applied generally. Finally, we compared the performance of three linkers, all of which are highly specific for the sulfhydryls of the reduced hinge of murine CC49, but differed in their stability under physiological conditions. A number of studies have shown that the nature of the linker can have a profound effect on the release of the drug once the ADC reaches the tumor. A vinyl sulfone linker was chosen for its resistance to proteolysis,^38^ while a bromoacetamide linker was chosen for its potential sensitivity to proteolysis likely to occur in the lysosome. A maleimide linker was chosen for its sensitivity to hydrolysis at physiological pH.^39^ Comparison of the first two linkers to maleimide was important, since maleimide‐based linkers have been reported to be degraded in the extracellular tumor microenvironment.^28^ However, maleimide‐based linkers can also be hydrolyzed while in circulation, leading to release of drug before the ADC reaches the tumor.^40^ Their performance was measured in vitro by cytotoxicity assays and in vivo by quantitative tumor targeting using PET imaging, and the reduced tumor growth seen in a xenograft model of ovarian cancer.

Since we had previously used a vinyl sulfone DO3A derivative conjugated to reduced hinge sulfhydryls of an antibody,^41^, ^42^ we began our study with a vinyl sulfone derivative of MMAE. As the reactivity of the methyl amine group of MMAE with vinyl sulfone was very low, it was necessary to perform the reaction in dry acetonitrile with an InCl_3_ catalyst, resulting in isolation of the VS‐MMAE in good yield. However, conjugation of VS‐MMAE to hinge reduced CC49 resulted in a maximum DAR of 2.3, suggesting that the efficiency of the reaction was low even at a 30 molar excess of VS‐MMAE over the antibody. Nonetheless, VS‐MMAE exhibited in vitro toxicity at three dose levels, and the in vivo tumor targeting of the VS‐MMAE conjugated antibody was not severely reduced compared to unconjugated antibody (17.3%ID/g vs 27.8%ID/g, 40 Hr postradiolabeled injection). When this ADC was tested at two dose levels for tumor reduction in a colon carcinoma xenograft model, there was no reduction or delay in tumor growth (Data not shown). Thus, this ADC was not selected for further studies. At this stage of development it was not clear if the ADC could be improved by increasing the DAR or by changing the linker to one that could better release the drug under physiological conditions.

To further study the effect of DAR and in vivo stability of linkers, we compared a bromoacetamido and a maleimido linker conjugated to the alpha‐methylamino group of MMAE. It should be noted that, unlike the sluggish reaction of VS, the reaction of bromoacetic anhydride with MMAE was fast and quantitative. The maleimide derivative was generated directly from bromoacetamido‐MMAE, simplifying the preparation of the two derivatives. In terms of in vitro and in vivo stability, the bromoacetamido derivative was expected to be stable,^43^ while the maleimido derivative was expected to slowly hydrolyze at pH 7.^28^, ^39^ Both derivatives gave high DARs, close to the theoretical of 10, based on the number of sulfhydryl's present in the reduced hinge of a murine IgG1. When the two derivatives were tested for in vitro cytotoxicity, the bromoacetamido‐MMAE ADC was cytotoxic at all doses tested while the maleimido‐MMAE was cytotoxic only at the highest dose.

Comparison between the pharmacokinetics of the bromoacetamido and maleimido linked ADC in tumor bearing mice revealed a major advantage of the bromoacetamido linked ADC, which retained about 6.43%ID/g vs 3.30%ID/g in the maleimido linked ADC in the tumors at 142 hours post radiolabeled injection. Compared to the VS‐MMAE ADC, the bromoacetamido linked ADC had identical pharmacokinetics, suggesting that the higher DAR of the bromoacetamido linked ADC had little effect. Based on this analysis, we expected better efficacy of the bromoacetamido linked ADC over the VS‐MMAE ADC due to the difference in DAR, and over the maleimido linked ADC due to the difference in tumor uptake.

This prediction was borne out in the treatment studies. The bromoacetamido linked ADC resulted in a significant delay in tumor growth and survival, compared to no significance seen for the maleimido linked ADC. We conclude that the maleimido linker is a poor choice for ADC‐based therapy even though it is capable of producing ADCs with a very high DAR. Unfortunately many ADCs in the field have a maleimide starting linkage, which has the potential to affect the efficacy of their ADC.^18^, ^20^ This paper found that the maleimide linkage was responsible for decreased potency of the ADC, due to the potential early release of the drug due to its instability at physiological pH. It should be noted that while tumor growth was delayed and survival increased with the acetamido linked ADC, no cures were observed, and the results were the same at the two highest doses, suggesting that no further improvement would be seen even with higher doses.

In comparison to other studies on CC49 ADCs, recently Rossini et al.^44^, ^45^ generated a series of Click reagent tagged MMAE ADCs that were administered four times followed by the complimentary Click agent to release MMAE at the site of the tumor. Instead of intact CC49, they selected a CC49 diabody‐based ADC to allow a more rapid biodistribution and the potential for further penetration into solid tumors.^46^ This rapid biodistribution is a requirement for two step antibody plus drug studies.^47^ The click chemistry used could also overcome the potential problem of noninternalizing antigen‐antibody complexes, as the released drugs can penetrate into the cell through passive diffusion.^48^ In their studies, the diabody click‐linked ADC alone increased the median survival by 25%, compared to our intact CC49‐Br‐MMAE that increased the median survival by 40%. However, when they repeated the study with the second Click agent to release the drug, tumor cures were observed. While the outcome was impressive, it is not clear how this “anti‐Click drug release” approach can be translated to a clinical setting, a major limitation to two step approaches. Nevertheless, the study is encouraging in that ADC treatment of solid tumors, while challenging, can be successful when properly optimized.

In terms of other anti‐mucin targeted ADCs utilizing MMAE, a recent study by Prendergast et al. showed impressive results for two of the ADCs tested.^9^ Although these studies were conducted in a breast cancer model, they further strengthen the ADC approach for solid tumors.

A critical aspect of any targeted therapy is the possibility of off‐target uptake. In this study, the uterus of the NSG mouse was shown to be is positive for TAG72 (Figure S5). Since the endometrium of human uterus is TAG72 positive,^31^ the NSG model may be ideal for testing the CC49 directed therapy. A further improvement of the mouse model would be to test for ADC efficacy in an IP tumor model. Preliminary studies have shown the untreated control mice can last almost 100 days until the mice need to be euthanized due to ascites fluid build‐up. Thus, the IP model in mice requires long‐term studies. If off‐target uptake occurs, predosing with the unconjugated antibody could be a potential solution. An unconjugated antibody dose may block unwanted expression of the antigens in normal tissue while still allowing for tumor targeting, when the antigen is more highly expressed on the cancer cells. Pretargeting with an unlabeled antibody before treatment is common practice in radioimmunotherapy, to prevent off ‐target, unwanted toxicity.^49^, ^50^ In addition to ovarian cancer, CC49 ADCs can be used in the treatment of other cancers since TAG72 is known to be expressed in breast, colon, pancreas, and prostate cancers, among other solid tumors.^3^, ^51^, ^52^, ^53^, ^54^

## 5 CONCLUSION

By investigating the importance of linker chemistry, we have shown that the stable linker of the bromoacetamido‐ADC was able to improve efficacy over a physiologically pH sensitive maleimide linked ADC, both with similar DARs. Based on the finding of this paper, switching the linker chemistry of ADCs with higher DARs, to more stable chemistry, could extend the half‐life of the ADC, in this model. The next step would be to further improve linker chemistry, taking into account the low internalization of the TAG72 antigen, while also considering how changing the linker stability can affect efficacy. In conclusion, a high DAR worked well for targeting and had an antitumor effect while maintaining tolerability in the mice, using the stable bromoacetamido linker chemistry.

## CONFLICT OF INTEREST

The authors declare that they have no known competing financial interests or personal relationships that could have appeared to influence the work reported in this paper.

## AUTHOR CONTRIBUTIONS

MM performed the experiments and wrote the first draft, LL and PY provided valuable reagents, JC performed PET imaging, EP radiolabeled samples, DC and JES proof read the ms

## Supporting information

Fig S1‐S9

## Data Availability

The data that support the findings of this study are available from the corresponding author (jshively@coh.org) upon reasonable request.
