# Supplementary material for: Improved targeting of an anti‐TAG‐72 antibody drug conjugate for the treatment of ovarian cancer
Source: Cancer Med. 2020 May 5;9(13):4756–67. doi: 10.1002/cam4.3078 (PMC7333846; doi:10.1002/cam4.3078)
Supplement: Supplementary file 1 — Fig S1‐S9 [file CAM4-9-4756-s001.docx]

**Improved targeting of an anti-TAG-72 antibody drug conjugate for the treatment of ovarian cancer**

Megan Minnix ^1,2^, Lin Li ^1^, Paul Yazaki ^1^, Junie Chea ^3^, Erasmus Poku ^3^, David Colcher ^1^ and John E. Shively ^1,*^

**Supplementary Figures**

**
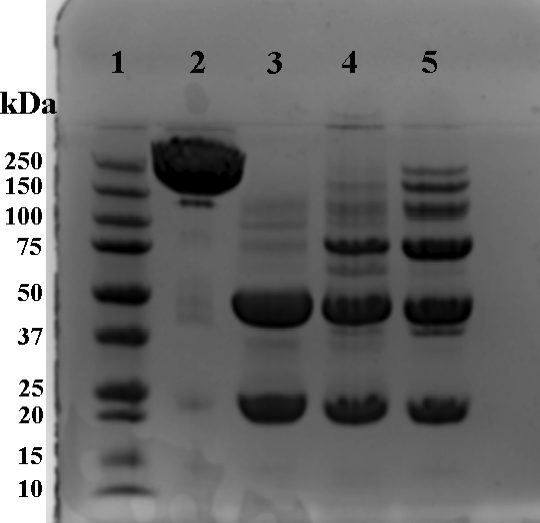
**

**Supplementary Figure S1. Analysis of ADCs by SDS gel electrophoresis**. Purified ADCs (5 µg) were analyzed by non-reducing SDS gel electrophoresis. **1**. Molecular mass markers. **2**. Murine CC49. **3**. Bromoacetamido-MMAE ADC. **4**. Maleimido-MMAE ADC. **5**. VS- MMAE-VS ADC. Bands at 50 and 25 kDa are heavy (H) and light (L) chains, respectively. Upper bands represent incompletely derivatized ADCs in which some HL (75 kDa), H_2_ (100 kDa) and H_2_L (125 kDa) species are formed. Integration of the H and L major bands for each product reveals >95% purity for each of the ADCs.

**
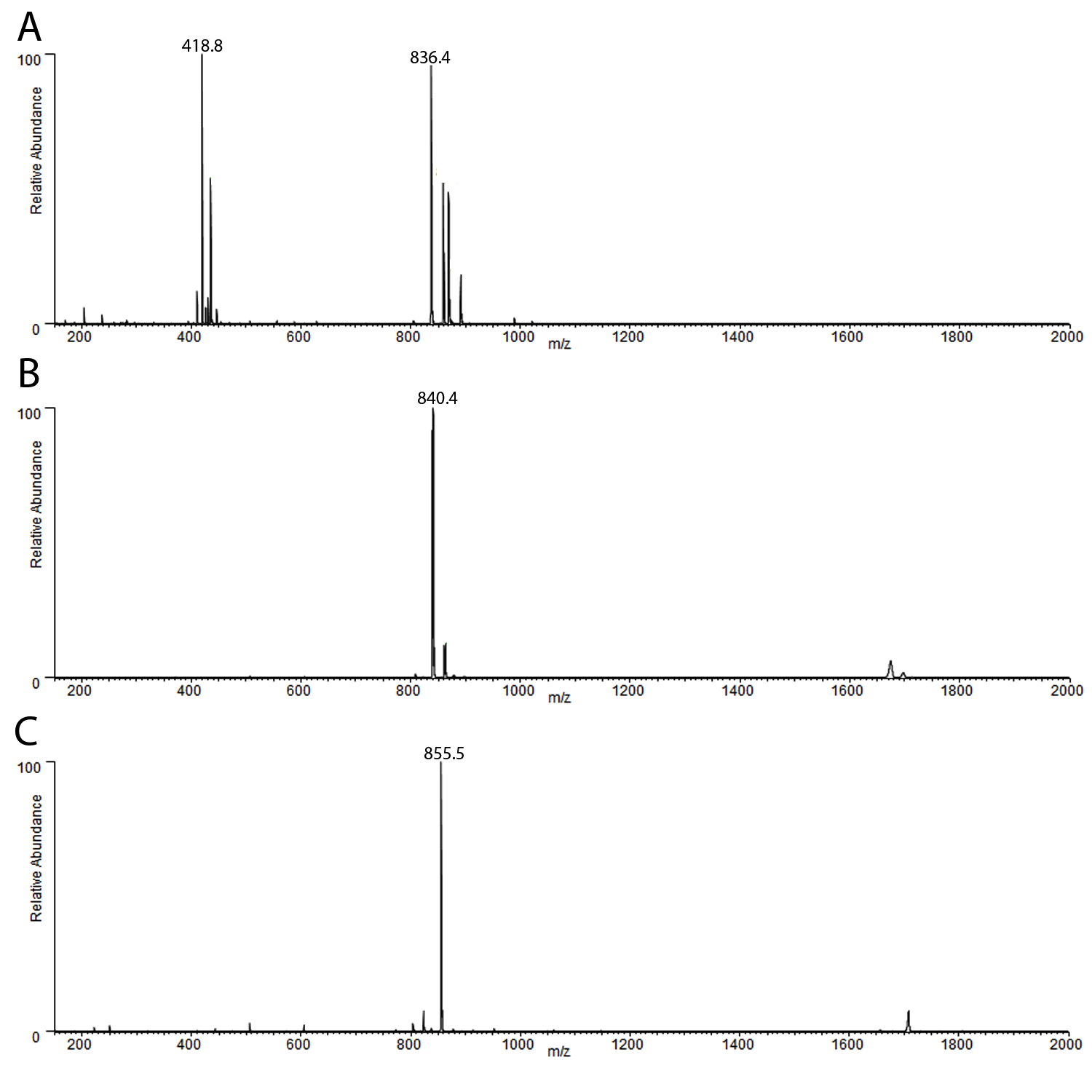
**

**Supplementary Figure S2. Analysis of MMAE-linkers by mass spectrometry**. RP-HPLC purified MMAE linkers were analyzed by mass spectrometry. **A**. MMAE-VS, MH^+^ obs. = 836.4, MH^+^ expected = 836.14. **B**. MMAE-bromoacetamido, MH^+^ obs. = 840.4, MH^+^ expected = 838.93. **C**. MMAE-mal, MH^+^ obs. = 855.5, MH^+^ expected = 855.09.


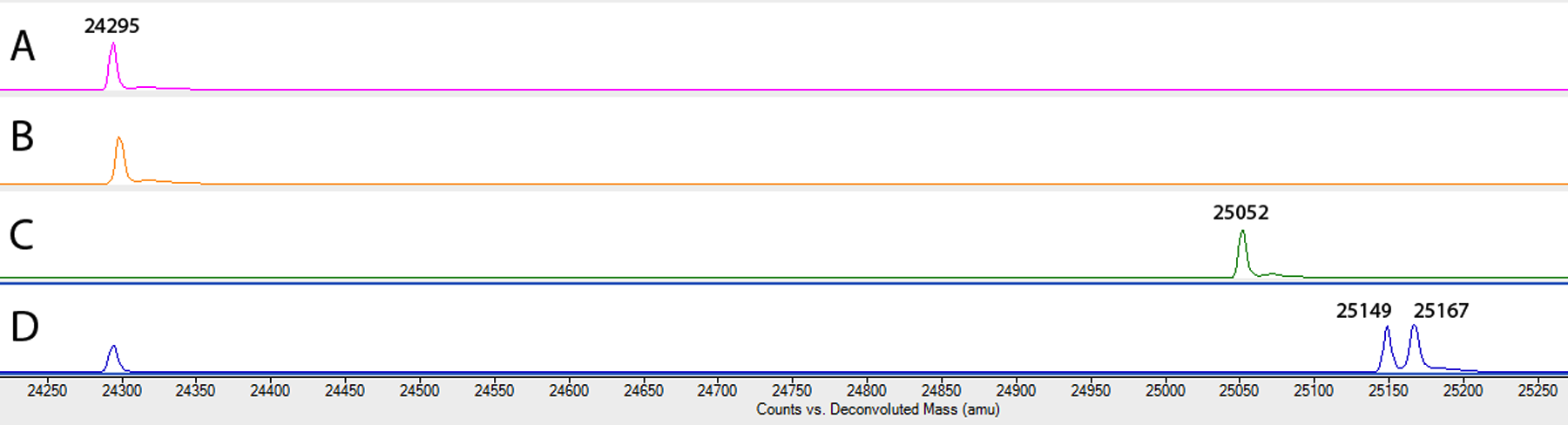


**Supplementary Figure S3. Q-TOF MS analysis of DAR on antibody light chains of ADCs.**

**A**. CC49 antibody. **B**. CC49-VS-MMAE. **C**. CC49-Br-MMAE. **D**. CC49-mal-MMAE.


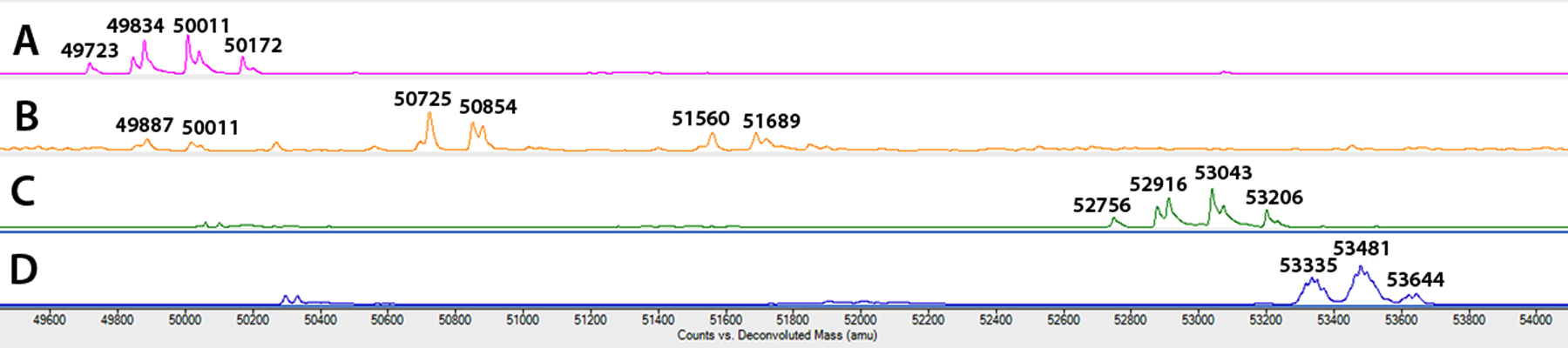


**Supplementary Figure S4. Q-TOF MS analysis of DAR on antibody heavy chains of ADCs.**

**A**. CC49 antibody. **B**. CC49-VS-MMAE. **C**. CC49-Br-MMAE. **D**. CC49-mal-MMAE.


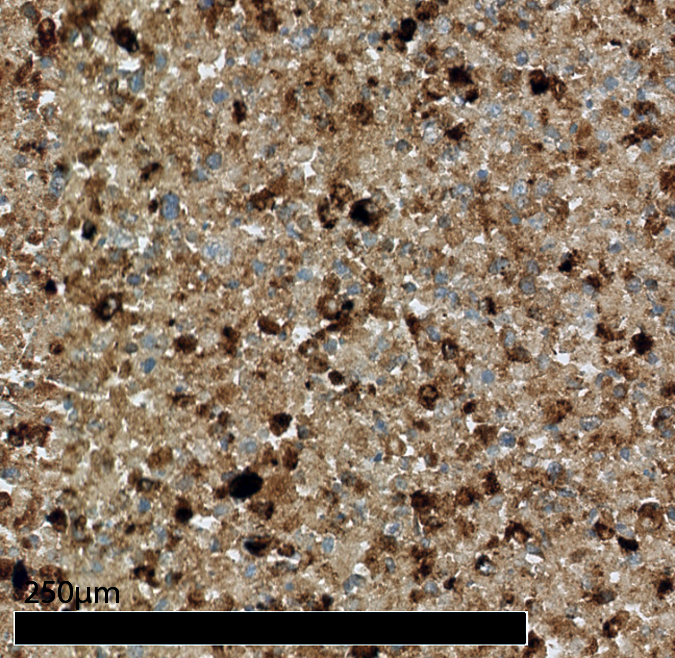


**Supplementary Figure S5. TAG72 Expression in tumor cystic fluid using Immunohistochemistry**.

Cystic tumor fluid from OVCAR3 xenografts in NSG mice was immonostained with muCC49.


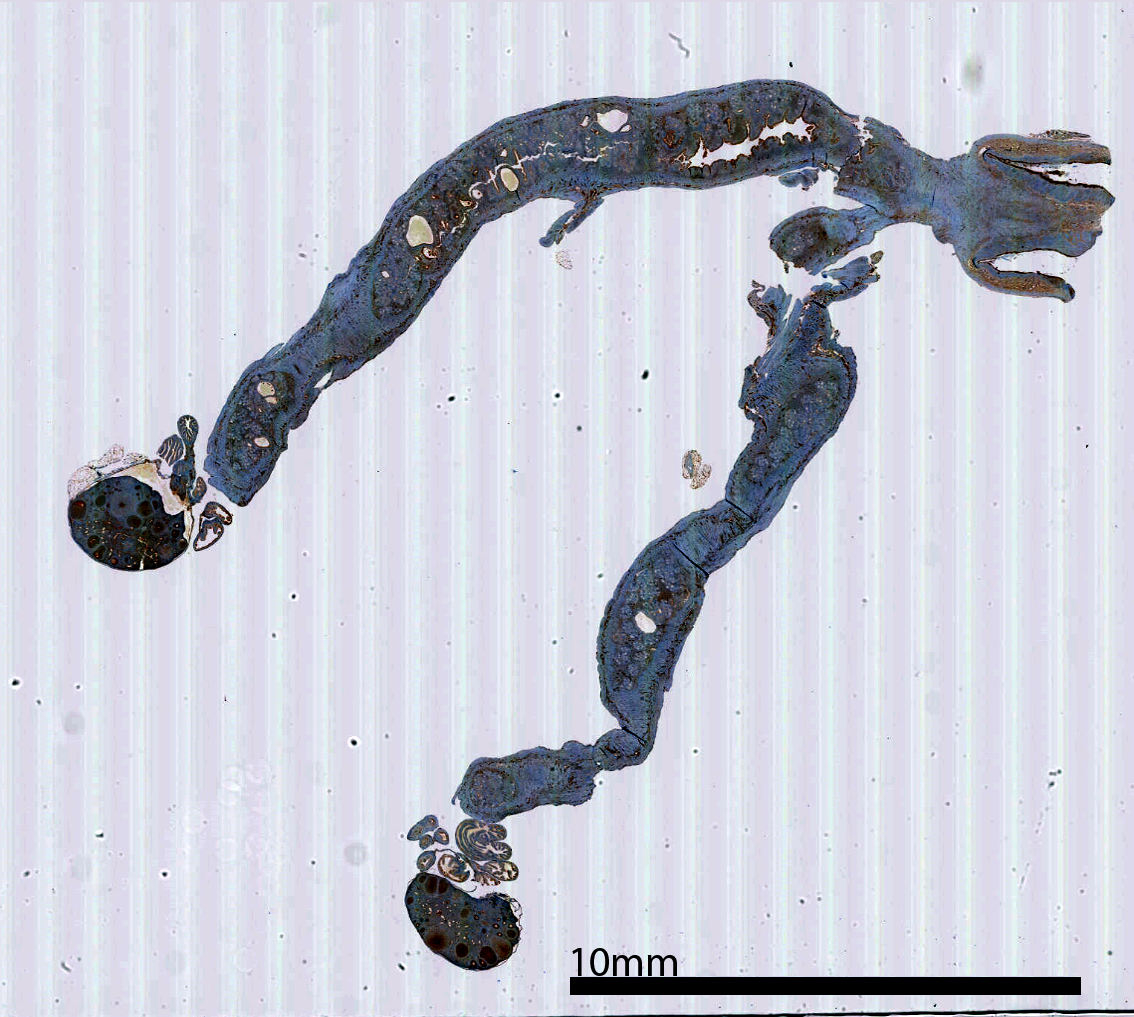


**Supplementary Figure S6. TAG72 Expression in uterus and ovary of NSG mice using Immunohistochemistry.** Uterus and ovaries collected from female NSG mice were immunostained with the CC49 antibody.


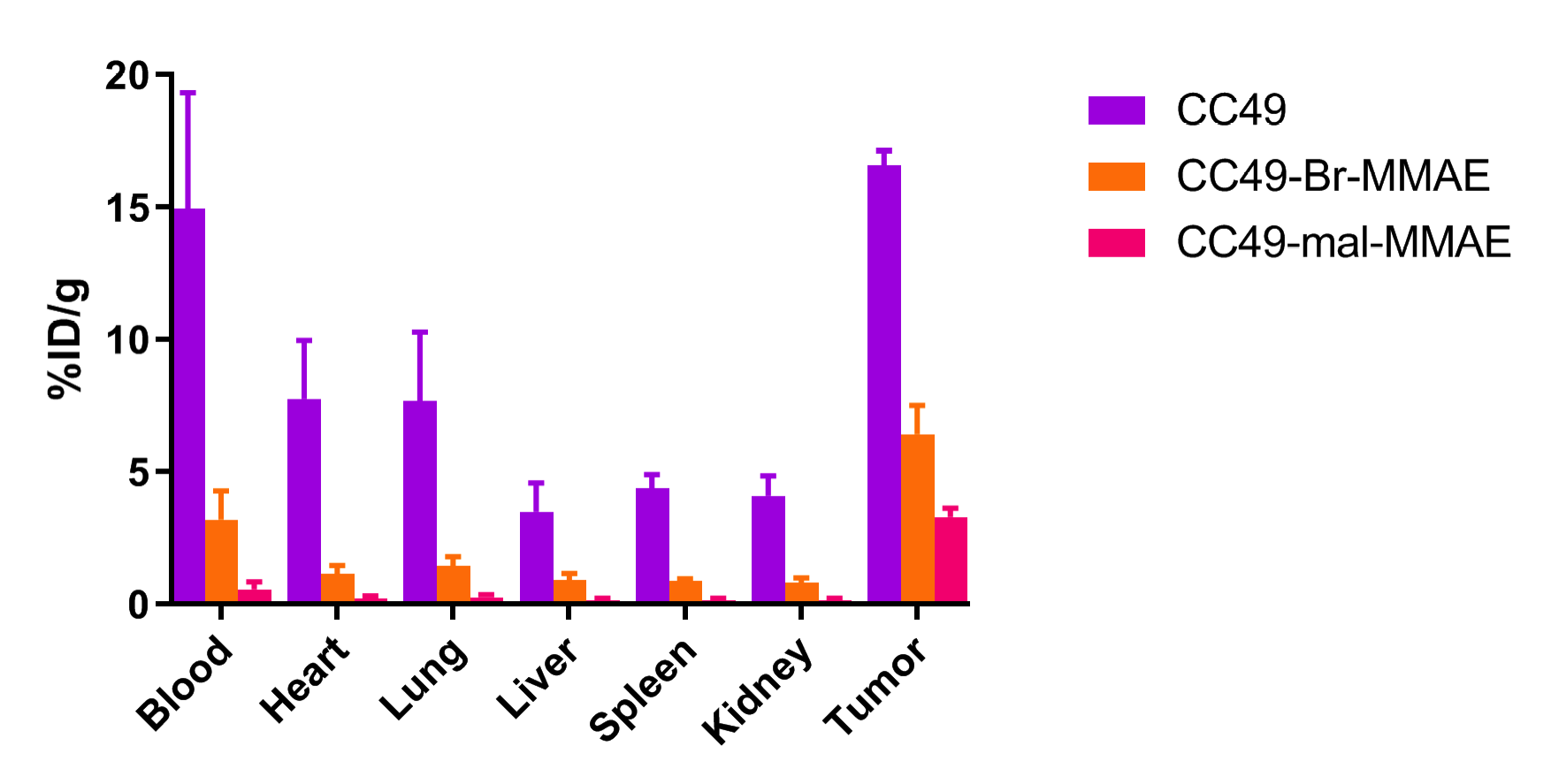


**Supplementary Figure S7. Terminal biodistributions of ^124^I-labeled CC49 antibody and ADCs in OVCAR3 xenografts in NSG mice at 142 hr post injection**. Radioiodinated CC49, CC49-Br-MMAE, or CC49-mal-MMAE were injected into NSG mice bearing OVCAR3 xenografts. Mice were euthanized at 142 hrs post injection and the %ID/g of tissues and blood was measured (n=2/group).


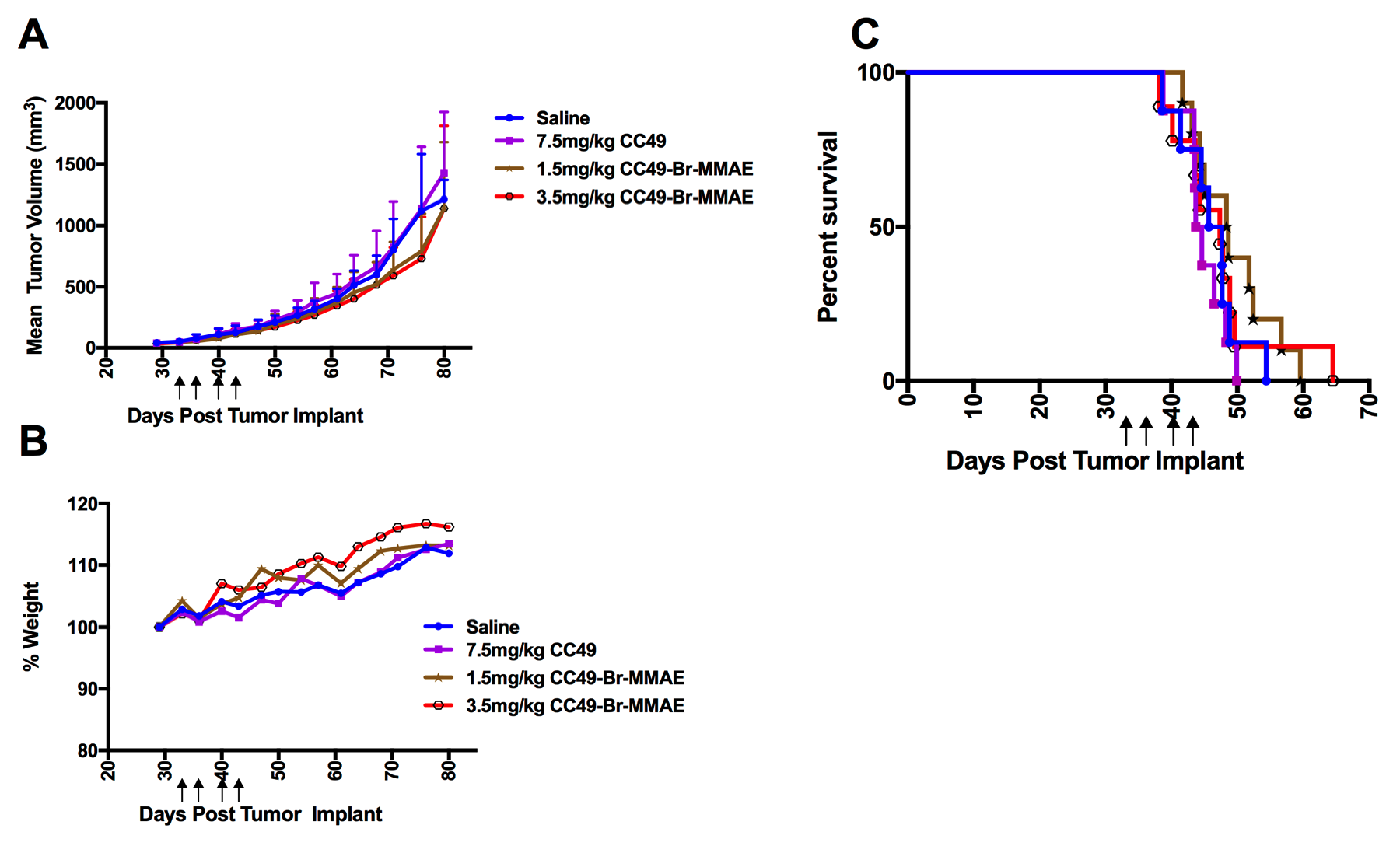


**Supplementary Figure S8. Mice treated with lower (1.5mg/kg and 3.5mg/kg) doses of CC49-Br-MMAE showed no reduction in tumor growth an no improvement in survival in the OVCAR3 xenograft model.** Female NSG mice with OVCAR3 xenografts were treated with CC49-Br-MMAE. Saline group (n=8), 7.5mg/kg CC49 antibody (n=8), 1.5mg/kg CC49-Br-MMAE (n=9), and 3.5mg/kg CC49-Br-MMAE (n=9). Mice were treated IP at days 33, 36, 40, and 43 post OVCAR3 injection. Black arrows represent time of doses. **A**. Tumor growth curves. The tumor growth curves of the CC49 antibody (p=0.994), 1.5mg/kg CC49-Br-MMAE (p=0.965) and 3.5mg/kg CC49-Br-MMAE (p=0.935) were not statistically significant compared to the saline control. **B**. Survival curves. The survival curves of the CC49 antibody (p=0.510), 1.5mg/kg CC49-Br-MMAE (p=0.241) and 3.5mg/kg CC49-Br-MMAE (p=0.659) were not statistically significant compared to the saline control. **C**. Weight loss of treated mice. The weight curves of the CC49 antibody (p=0.999), 1.5mg/kg CC49-Br-MMAE (p=0.718) and 3.5mg/kg CC49-Br-MMAE (p=0.213) were not statistically significant compared to the saline control.


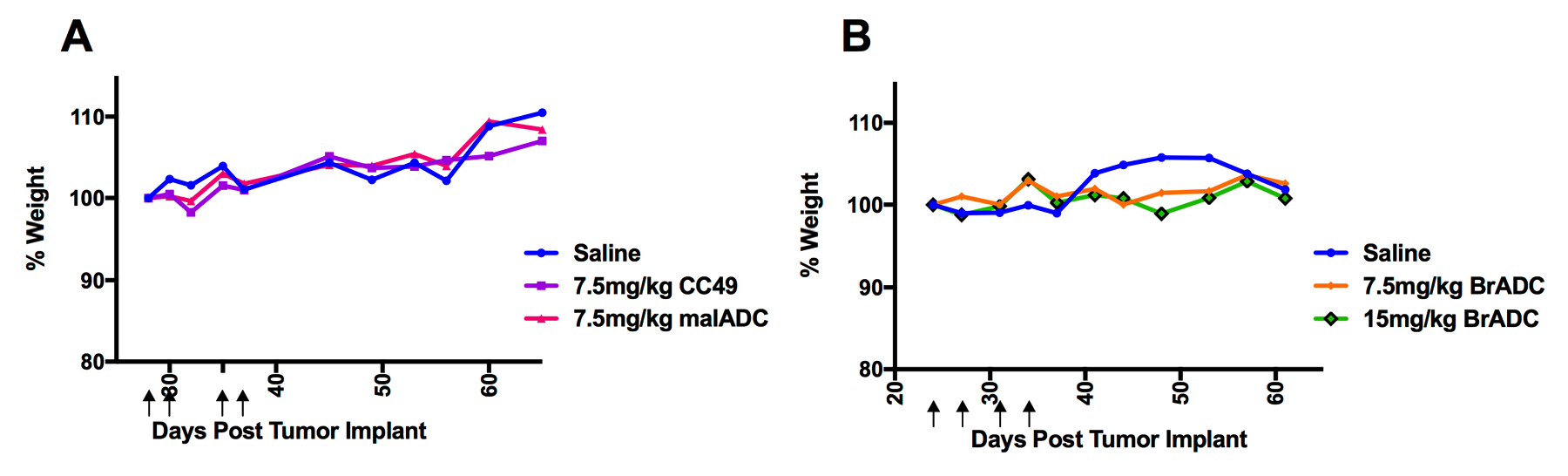


**Supplementary Figure S9. Weight loss of mice treated with ADCs in OVCAR3 xenograft model.**

Female NSG mice earing OVCAR3 xenografts were treated with 4 IP doses of indicated ADCs and their weights recorded over time. **A**. CC49-mal-MMAE treated mice at days 28, 30, 35, and 37 post OVCAR3 injection. Saline (n=8), CC49 (n=10), and CC49-mal-MMAE (n=10). **B**. CC49-Br-MMAE treated mice at days 24, 27, 31, and 34 post OVCAR3 injection. Saline (n=4), 7.5mg/kg CC49-Br-MMAE (n=8), 15mg/kg CC49-Br-MMAE (n=8). There were no statistical differences in any of the weight curves using one way ANOVA.
